# ROS Suppression by Egg White Hydrolysate in DOCA-Salt Rats—An Alternative Tool against Vascular Dysfunction in Severe Hypertension

**DOI:** 10.3390/antiox11091713

**Published:** 2022-08-30

**Authors:** Edina da Luz Abreu, Camila Rodrigues Moro, Samia Hassan Husein Kanaan, Ricardo Bernardino de Paula, Camila Teixeira Herrera, Pedro Henrique Dorneles Costa, Franck Maciel Peçanha, Dalton Valentim Vassallo, Luciana Venturini Rossoni, Marta Miguel-Castro, Giulia Alessandra Wiggers

**Affiliations:** 1 Cardiovascular Physiology Laboratory, Universidade Federal do Pampa, BR 472, Km 592, Uruguaiana 97501-970, Brazil; 2 Department of Physiology and Biophysics, Institute of Biomedical Science, University of São Paulo, Av. ProfessorLineu Prestes, nº 2415, São Paulo 97501-970, Brazil; 3 Cardiac Electromechanical and Vascular Reactivity Laboratory, Universidade Federal do Espírito Santo, Av. Marechal Campos, 1468, Vitória 29040-090, Brazil; 4 Instituto de Investigación en Ciencias de la Alimentación (CIAL, CSIC-UAM.), C/Nicolás Cabrera, 9, Campus Universitario de Cantoblanco, 28049 Madrid, Spain

**Keywords:** egg white hydrolysate, hypertension, deoxycorticosterone acetate (DOCA)-salt, oxidative stress, inflammation, mitochondria

## Abstract

This study aimed to evaluate the potential for lowering blood pressure and beneficial effects on mesenteric resistance arteries (MRA) and conductance vessels (aorta) produced by dietary supplementation of an egg white hydrolysate (EWH) in rats with severe hypertension induced by deoxycorticosterone plus salt treatment (DOCA-salt), as well as the underlying mechanisms involved. The DOCA-salt model presented higher blood pressure, which was significantly reduced by EWH. The impaired acetylcholine-induced relaxation and eNOS expression observed in MRA and aorta from DOCA-salt rats was ameliorated by EWH. This effect on vessels (MRA and aorta) was related to the antioxidant effect of EWH, since hydrolysate intake prevented the NF-κB/TNFα inflammatory pathway and NADPH oxidase-induced reactive oxygen species (ROS) generation, as well as the mitochondrial source of ROS in MRA. At the plasma level, EWH blocked the higher ROS and MDA generation by DOCA-salt treatment, without altering the antioxidant marker. In conclusion, EWH demonstrated an antihypertensive effect in a model of severe hypertension. This effect could be related to its endothelium-dependent vasodilator properties mediated by an ameliorated vessel’s redox imbalance and inflammatory state.

## 1. Introduction

Hypertension is one of the most common chronic diseases worldwide. It is of multifactorial origin and results from a complex interaction between environmental (such as dietary) and genetic factors [1,2,3]. Although a larger number of pharmacological and non-pharmacological therapies are used, some patients are resistant to treatment, mainly those who present severe hypertension [2,4]. Thus, more studies are necessary to improve blood pressure control in hypertension. Interestingly, endothelial dysfunction is a hallmark of hypertension, and the resistance and conductance arteries are crucial for the regulation and maintenance of blood pressure [3]. In hypertension, oxidative stress is implicated in vascular injuries [5,6,7], and the main vascular sources of oxidative stress are the excessive production of superoxide anion (O_2_^−^) by NADPH oxidase, xanthine oxidase, mitochondria, cyclooxygenase-2, and/or uncoupled nitric oxide (NO) synthase [6,7,8]. As a result of this oxidative stress, endothelium-dependent vasodilation is impaired, and vascular stiffness and peripheral vascular resistance are increased, contributing to the development and maintenance of arterial hypertension, leading to damage to end-organs [5,6,9].

Considering its complexity, hypertension is studied in different models of experimental rodents, including deoxycorticosterone acetate (DOCA)-salt hypertension, a volume-dependent hypertension model that combines DOCA treatment, and high salt intake with uninephrectomy. This model induces severe hypertension and impacts blood volume, cardiac output, and peripheral vascular resistance; thus, it is a helpful model for studies on resistant hypertension [7,10,11].

Non-pharmacological strategies to prevent or treat hypertension have gained attention. In this context, natural bioactive compounds from foods seem safe and low-cost alternatives. Moreover, this strategy generally does not present side effects common in the polytherapy usually used for the treatment of hypertension [4,12]. Among them, bioactive peptides derived from food proteins with antihypertensive and antioxidant properties have been reported to be successfully used to control hypertension and related disorders. In previous works, enzymatic hydrolysis of egg white proteins with pepsin for eight hours produced multifunctional peptides with angiotensin-converting enzyme (ACE) inhibitory action and antioxidant, anti-inflammatory, and vasorelaxant activities [13,14,15,16,17,18,19,20,21,22,23,24]. A blood- pressure-lowering effect related to ACE inhibition and antioxidant activity was also demonstrated after short- and long-term administration of this hydrolysate on a genetic model of hypertension––spontaneously hypertensive rats (SHR) [14,15]. However, the effect and mechanisms of this functional food administration on blood pressure and vascular dysfunction in the severe hypertension model have been little explored.

This study aimed to evaluate the potential antihypertensive and vasculoprotective effects of dietary supplementation of an egg white hydrolysate (EWH) in DOCA-salt hypertensive rats and the mechanisms involved.

## 2. Material and Methods

### 2.1. Egg White Hydrolysate Preparation

EWH was obtained by enzymatic hydrolysis of raw egg whites after treatment with food-grade pepsin, as described previously by Garcés-Rimón et al., 2016 [20]. For eight hours, pasteurized egg white was hydrolyzed with pepsin BC 1:3000 (Biocatalyst, UK). Enzyme inactivation was achieved by increasing the pH to 7.0 with NaOH (5 N). The hydrolysate was centrifuged at 2500× *g* for 15 min, and the supernatant was frozen and lyophilized until used.

### 2.2. Animals and Treatment

Male Wistar rats (180–220 g) were obtained from the Central Animal Laboratory of the Federal University of Pelotas (Rio Grande do Sul, Brazil). Animals were maintained at standard conditions (constant room temperature, humidity, and 12:12 h light-dark) with water and fed *ad libitum* in the Federal University of Pampa vivarium. The experimental protocols were performed according to guidelines of the National Council of Ethics with Animals (CONCEA) and National Institute of Health Guide for the Care and Use of Laboratory Animals (NIH, 1996), and the Local Institution Animal Care and Use Committee (protocol number 003/2020).

Rats were submitted to the tail-cuff plethysmography to evaluate the systolic blood pressure (SBP) levels before the uninephrectomy (time −1/week) and the induction of the hypertensive model. Afterward, all animals were submitted for right kidney removal as previously described [7]. Thus, the rats were anesthetized (64.9 mg/kg ketamine, 3.2 mg/kg xylazine, and 0.78 mg/kg acepromazine, *i.p.*) and underwent a small incision in the right flank for kidney removal. All animals received analgesia for 3 days post-surgery (Ketofen^@^ 0.2 mg/kg, *s.c.*).

**Figure 1:**
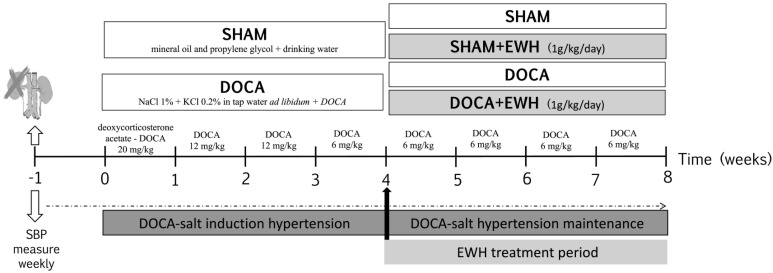
Schematic representation of experimental groups, DOCA-salt hypertension induction model and EWH treatment period.

As observed in Figure 1, seven days after surgery (time 0), rats were randomly divided into two groups: DOCA-salt and SHAM (Vehicle). Animals of the SHAM group received vehicle injections (1:1 mineral oil and propylene glycol) and drinking water. Animals of the DOCA-salt group received subcutaneous injections of DOCA (Sigma Aldrich, Darmstadt, Germany) diluted in 1:1 mineral oil and propylene glycol at the sequential doses of 20 mg/kg in the first week, 12 mg/kg in the second and third week, and 6 mg/kg from the fourth week to the eighth week of the experimental period, plus water supplemented with 1.0% of NaCl and 0.2% of KCl [7]. In the last four weeks of the experimental period, the groups were subdivided into animals treated with EWH diluted in water (1 g/kg/day, by gavage) [21] or water, by gavage. Thus, the animals constituted 4 experimental groups: SHAM, SHAM+EWH, DOCA, and DOCA+EWH.

### 2.3. Systolic Blood Pressure Measurement

As described above, SBP was measured weekly using a standard non-invasive tail-cuff plethysmography method (Pneumatic transducer, AD Instruments Pty Ltd., Bella Vista, NSW, Australia) in conscious, restrained rats as described [25]. Briefly, rats were heated to 37 °C for 10 min to detect caudal artery pulsations. Afterward, the transducer was placed on the tail, and the average of ten collected times of SBP was used. To ensure the reliability of the measurements, all animals were previously acclimatized, and measurements were made for the same person at the same time of day. The measurements were made weekly in the following times: time −1 and 0, before, and 1 week after uninephrectomy; time 1 to 4, 1 to 4 weeks after DOCA-salt or SHAM treatment; and time 4–8, after the co-treatment with EWH or water (Figure 1).

### 2.4. Blood, Tissue Collection, and Mesenteric and Aorta Vascular Reactivity Experiments

At the end of the experimental period, animals were anesthetized (87 mg/kg ketamine, and 13 mg/kg xylazine, *i.p.*) and euthanized. Blood samples, mesenteric resistance arteries (MRA), and thoracic aorta were removed. Arteries were cleaned of connective tissue, and some rings were inserted in a freezing medium (OCT) and frozen at −80 °C.

For vascular reactivity experiments, third order MRA (2 mm) and thoracic aorta (4 mm) segments with (E+) and without endothelium (E-, mechanically removed) were mounted in a wire myograph for small and larger vessels [26,27]. After 45 min of equilibration period in gassed (95% O_2_ and 5% CO_2_) Krebs–Henseleit solution (in mM: 115 NaCl, 25 NaHCO_3_, 11.1 glucose, 4.7 KCl, 2.5 CaCl_2_, 1.2 MgSO_4_·7H_2_O, 1.2 KH_2_PO_4_, and 0.01 Na_2_EDTA; pH 7.4, 37 °C), MRA were stretched to their optimal lumen diameter to develop active tension, and aorta segments maintaining an optimal resting tension of 1.5 g. MRA diameter were similar among groups (SHAM: 292.2 ± 4.8 vs. SHAM+EWH: 291.1 ± 8.4 vs. DOCA: 288.5 ± 8.2 vs. DOCA+EWH: 291.1 ± 4.5 µm, n = 10; Two-way ANOVA, *p* > 0.05).

To verify the smooth muscle integrity and the maximal tension developed, MRA and aorta segments were exposed twice to a high-potassium solution (KPSS-120 or 75 mM, respectively). The vascular response to KPSS remained unaltered in MRA (SHAM: 4.0 ± 0.1 vs. SHAM+EWH: 4.2 ± 0.2 vs. DOCA: 4.0 ± 0.1 vs. DOCA+EWH: 4.3 ± 0.1 mN/mm, n = 10; Two-way ANOVA, *p* > 0.05) and in aorta (SHAM: 1.5 ± 0.1 vs. SHAM+EWH: 1.4 ± 0.1 vs. DOCA: 1.5 ± 0.1 vs. DOCA+EWH: 1.7 ± 0.1 g, n = 10; Two-way ANOVA, *p* > 0.05) among groups.

After 60 min, the endothelium-dependent relaxation to acetylcholine (ACh–Sigma-Aldrich, St. Louis, MO, USA) was assessed. MRA and aorta segments were pre-contracted with α-adrenergic agonists (norepinephrine–NE and phenylephrine–Phe, respectively; Sigma-Aldrich), in a concentration that produced tension close to 50% of those induced by KPSS and a concentration–response curve to ACh (1 nM–1 mM) was performed. To evaluate the endothelium-independent relaxation, concentration–response curves to NO donor sodium nitroprusside (SNP, 0.1 nM–300 µM; Sigma-Aldrich) were performed in NE or Phe pre-contracted segments.

To evaluate the participation of NO, reactive oxygen species (ROS), mitochondrial superoxide anion production, NF-κB activity on ACh-induced relaxation, and some MRA and/or aorta segments with intact endothelium (E+) were pre-incubated for 30 min with: (*i*) Nω-nitro-L-arginine methyl ester (L-NAME, 100 μM; Cat. Nº N5751—Sigma-Aldrich), a non-selective NO synthase (NOS) inhibitor (only in the aorta); (*ii*) superoxide dismutase (SOD, 750 U/mL; Cat. Nº s5395—Sigma-Aldrich), superoxide anion scavenger; (*iii*) MitoTEMPO (0.5 µM; Cat. Nº SML0737—Sigma-Aldrich), specific scavenger of mitochondrial O_2_^−^; and (*iv*) BAY 117,082 (5 µM; Cat. Nº B5556- Sigma-Aldrich) NF-κB inhibitor (only in MRA), respectively.

### 2.5. Determination of Oxidative Stress Biomarkers in Plasma, MRA, and Aorta

To evaluate the oxidative stress biomarkers in MRA and aorta, the vessels were homogenized in Tris-HCl (50 mM, pH 7.4) and centrifuged at 2400× *g* for 10 min at 4 °C, and the supernatant fraction was used.

Malondialdehyde levels (MDA), a measure of lipid peroxidation, were performed using the colorimetric method. Briefly, thiobarbituric acid (TBA) 0.8%, acetic acid buffer (pH 3.2) plus sodium dodecyl sulfate (SDS) 8% were added to the samples, and the color reaction was measured against blanks (532 nm—SpectraMax M5 Molecular Devices, San Jose, CA, USA). The results were expressed as nmol MDA/g tissue [28].

ROS levels [29] were determined by 2′, 7′-dichlorofluorescein diacetate (DCF-DA) using the spectrofluorometric method. Briefly, after being diluted (1:5 in Tris-HCl 50 mM; pH 7.4), samples received 2′, 7′-dichlorofluorescein diacetate (DCF-DA, 1 mM; Cat. Nº 6883—Sigma-Aldrich). The DCF fluorescence intensity emission represents the amount of ROS formed (520 nm emission with 480 nm excitation for 60 min at 15 min intervals––SpectraMax M5 Molecular Devices, San Jose, CA, USA). The ROS levels were expressed as fluorescence units.

The total antioxidant capacity (FRAP) was measured by the Ferric Reducing/Antioxidant Power (FRAP) assay [30]. Briefly, the sample was added to FRAP reagent 10:1:1 [Sodium acetate buffer—300 mM pH 3.6; 2,4,6-Tri(2-pyridyl)-s-triazine– TPTZ—10 mM (Cat. Nº 93285, Sigma-Aldrich); Iron (III)—FeCl_3_—20 mM (Cat. Nº 157740, Sigma-Aldrich)] and incubated at 37 °C for 10 min. The absorbance was read at 593 nm (SpectraMax M5 Molecular Devices, San Jose, CA, USA). Data are expressed in reference to mM Trolox equivalents.

### 2.6. In Situ Detection of Vascular O_2_^−^ Production in MRA and Aorta and NO Production in the Aorta

O_2_^−^ production in situ in MRA and aorta was measured by dihydroethidium (DHE) (Cat. Nº D2310, Invitrogen Life Technologies, Waltham, MA, USA), as previously described by Piech et al. (2003) [31]. To evaluate the source of the O_2_^−^, mitochondrial and/or by the antioxidant activity of superoxide dismutase (SOD), some sections were incubated with DHE plus Mito-Tempo a scavenger of mitochondrial superoxide anion (0.5 mM—Sigma Aldrich) or MnTMPyP, a mimetic of SOD (25 μM; Cat. Nº ALX-430-07—Enzo Life Sciences, Farmingdale, NY, USA), respectively. The mean fluorescence densities were calculated using NIH ImageJ software V1.56 (National Institutes of Health, Bethesda, MD, USA) using the same imaging settings in each case. The results are expressed as arbitrary units (AU) of fluorescence intensity.

NO production was measured in the aorta sections using the NO-sensitive fluorescent dye 4,5-diaminofluorescein diacetate (DAF-2; Cat. Nº D225, Sigma Aldrich), according to Couto et al. (2015) [32]. The aortic sections were equilibrated for 10 min in phosphate buffer (0.1 mol/L, pH 7.4) that contained CaCl_2_ (0.45 mmol/L). The fresh buffer that contained DAF-2 (8 μmol/L) was topically applied to each tissue section and incubated in a light-protected humidified chamber at 37 °C. After 25 min of incubation, sections of each artery were stimulated without (Basal) or with ACh (100 μmol/L) for 15 min. The concentration of ACh used to evaluate the NO production in the aorta was selected based on functional vascular experiments. The images were analyzed with Image J software using the integrative density of the fluorescence observed in the artery in relation to the background staining in sections with and without Ach stimulation.

### 2.7. Immunofluorescence in MRA and Aorta

Arterial segments of MRA and aorta were prepared and analyzed according to Jimenez-Altayó et al. (2005) [33]. The primary antibodies used were against *NOX1* (1:400; Cat. 2108601, Sigma Aldrich), *eNOS* (1:400; Cat. SAB4502615, Sigma Aldrich), *NF-kB* (1:400; Cat. 4502615, Sigma Aldrich), and *TNFα* (1:400; Cat. 5700627, Sigma Aldrich). Alexa 488-conjugated goat anti-mouse immunoglobulin G [IgG]) was diluted at 1:400 (Cat. Nº A11001, Invitrogen Life Technologies). DAPI (1:500; Cat. MBD0015, Sigma Aldrich) to stain nuclei was used. In the preparation of negative control sections, we omitted the primary antibody. Images were acquired using an EVOS^®^ Floid^®^ Cell Imaging Station (Life Technologies, Carlsbad, CA, USA). For quantification, sections with the same capture parameters were analyzed. The mean fluorescence densities (histogram) using ImageJ were calculated. Data are expressed as fluorescence intensity.

### 2.8. Statistical Analysis

Data are expressed as the mean ± SEM. Vasodilator responses to ACh or SNP were expressed as a percentage of relaxation of the pre-contraction induced by NE or Phe. Results were analyzed using two-way ANOVA followed by a post hoc Bonferroni test (GraphPad Prism 8 software, San Diego, CA, USA). Differences were considered statistically significant with values of *p* < 0.05.

## 3. Results

**Figure 2:**
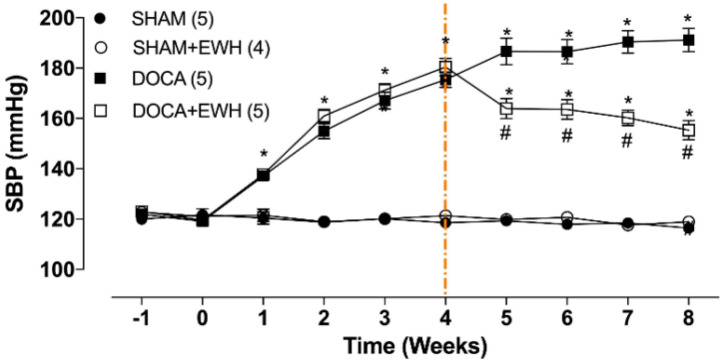
Effect of EWH on SBP in DOCA-salt hypertensive rats. SBP values of SHAM, SHAM+EWH, DOCA, and DOCA+EWH groups, measured before and after nephrectomy (−1 and 0 weeks), during DOCA-salt model induction (1 to 4th week), and during the maintenance of the DOCA-salt hypertensive model co-treated or not with EWH (5th to 8th week). The EWH treatment started on the 4th week after DOCA-salt or SHAM treatment (orange-marked line). Data are expressed as mean ± SEM. The number of rats is indicated in parentheses. Two-way ANOVA followed by Bonferroni post-test: *p* < 0.05 * vs. SHAM; # vs. DOCA.

SBP was similar among groups before starting the experimental period (Figure 2, time −1), and the uninephrectomy did not alter this parameter (Figure 2, time 0). As expected, DOCA-salt treatment progressively increased SBP from week 1 to week 4 (Figure 2, time 1–4). After the 4th week of treatment, DOCA animals maintained high blood pressure levels as compared to SHAM, but DOCA+EWH animals present lower SBP values than DOCA animals (Figure 2, time 4–8). This reduction was 36% compared to the untreated DOCA group, without reaching the SBP values of the SHAM group (Figure 2). SHAM+EWH animals maintained blood pressure levels similar to those observed in SHAM animals (Figure 2).

**Figure 3:**
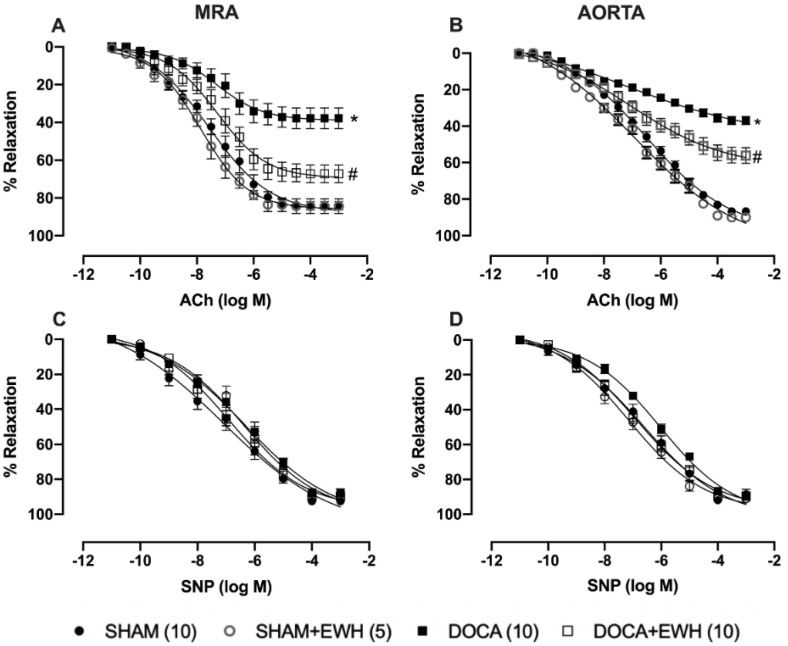
Effect of EWH in endothelium-dependent and -independent relaxation of MRA and aorta segments from DOCA-salt rats. Concentration–response curves to ACh (**A**,**B**) and SNP (**C**,**D**) in MRA and aorta segments from SHAM, SHAM+EWH, DOCA, and DOCA+EWH groups. The results are expressed (mean ± SEM) as the percentage of relaxation responses in norepinephrine or phenylephrine precontracted rings. The number of rats is indicated in parentheses. Two-way ANOVA followed by Bonferroni post-test: *p* < 0.05 * vs. SHAM; # vs. DOCA.

The endothelium-dependent relaxation induced by ACh, in both arteries, was reduced in the DOCA group as compared to SHAM (Figure 3A,B). EWH co-treatment improved ACh-induced relaxation in MRA and aorta of DOCA animals and did not change it in SHAM animals (Figure 3A,B). The SNP-induced relaxation was not altered in both vessels among groups (Figure 3C,D).

**Figure 4:**
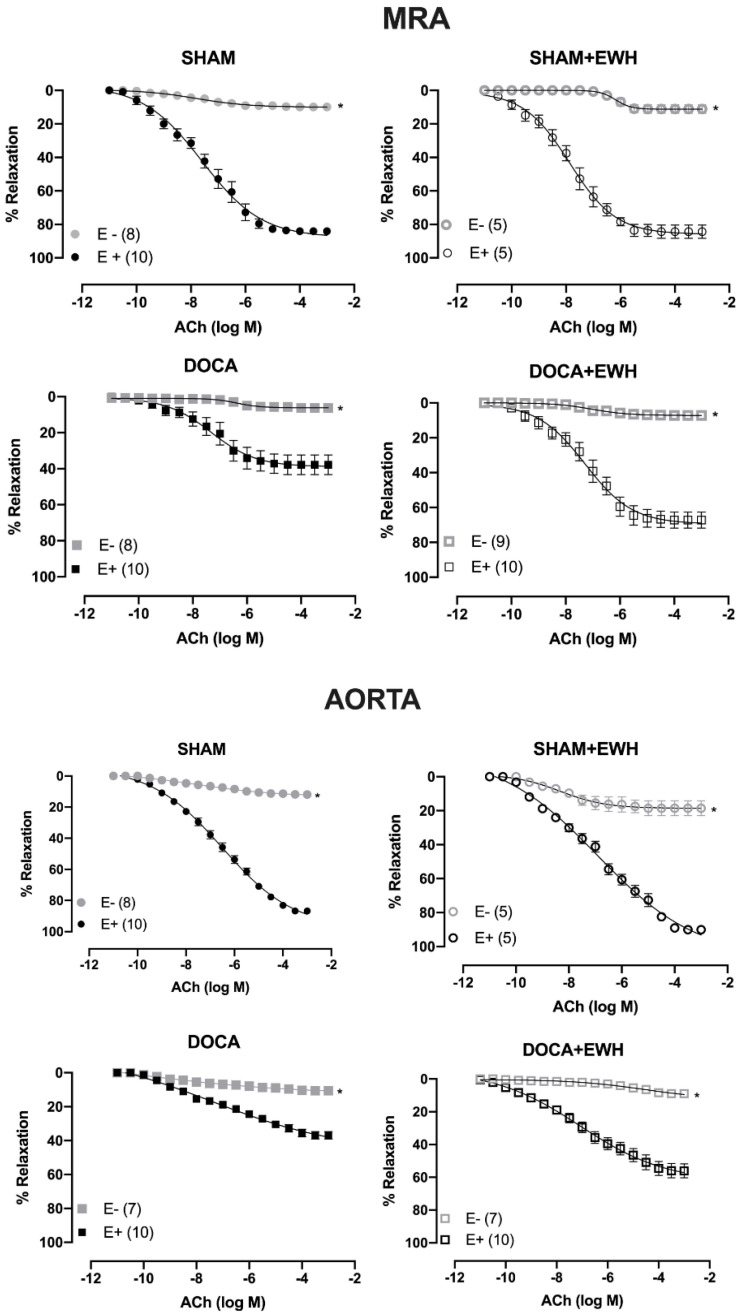
The role of endothelium on the effect of EWH in ACh-induced relaxation of MRA and aorta segments from DOCA-salt rats. Concentration–response curve to ACh in the presence (E+) and absence of endothelium (E−) in MRA (Upper graphics) and aorta (Bottom graphics) segments from rats SHAM, SHAM+EWH, DOCA, and DOCA+EWH. The results are expressed (mean ± SEM) as the percentage of relaxation responses in norepinephrine or phenylephrine precontracted rings. The number of rats is indicated in parentheses. Two-way ANOVA followed by Bonferroni post-test: *p* < 0.05 * vs. E+.

The removal of the endothelium (E-) reduced the ACh-induced relaxation in MRA and aorta of all groups as compared to the respective E+ segments (Figure 4). The role of the endothelium in the relaxation response to ACh was strongly reduced in MRA and aorta of DOCA animals compared to SHAM, whereas EWH co-treatment partially restores this response in both arteries (Figure 4).

**Figure 5:**
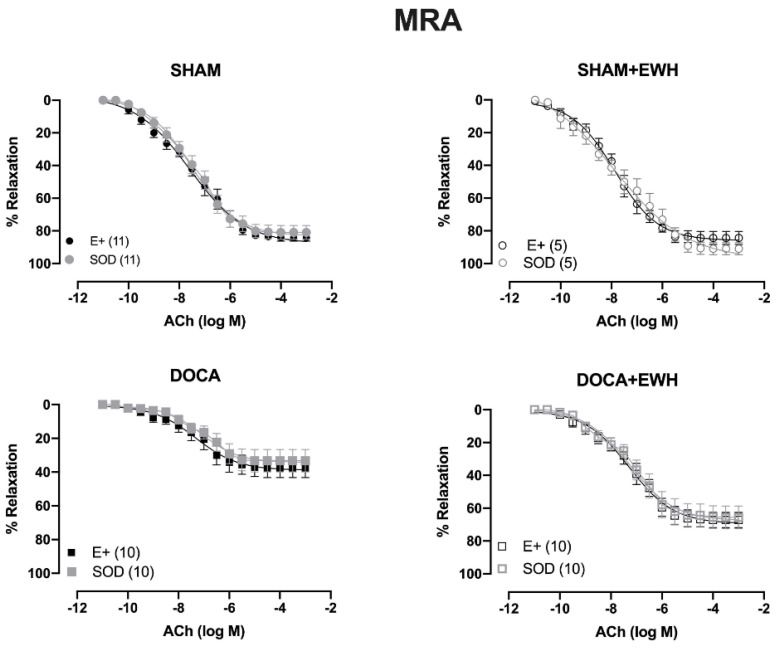

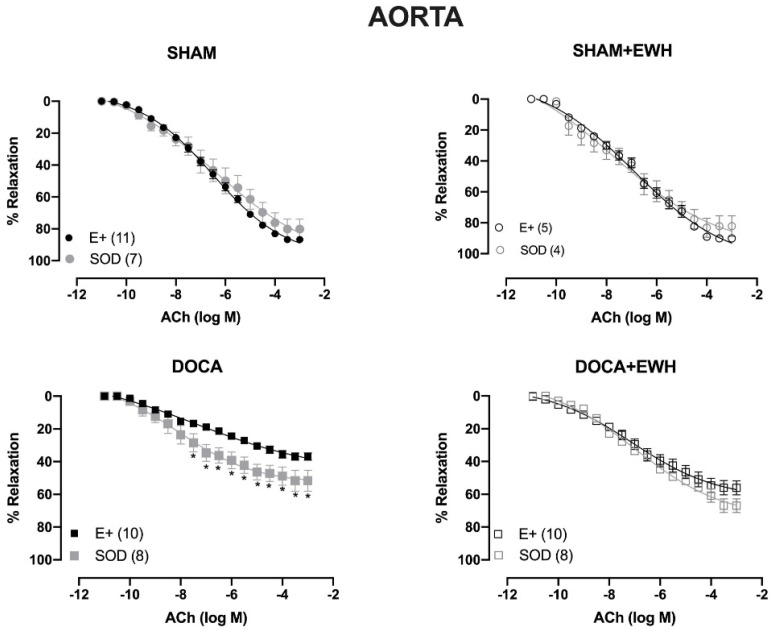
The role of EWH in ROS-mediated ACh-induced relaxation in MRA and aorta of DOCA-salt rats. Concentration–response curves to ACh were obtained in MRA (Upper graphics) and aorta (Bottom graphics) segments from rats from SHAM, SHAM+EWH, DOCA, and DOCA+EWH groups before (E+) and after incubation with scavenger reactive oxygen species, the superoxide dismutase (SOD, 750/mL). The results are expressed (mean ± SEM) as the percentage of relaxation responses in norepinephrine or phenylephrine precontracted rings. The number of animals in each group is in parentheses. Two-way ANOVA followed by Bonferroni post-test: *p* < 0.05 * vs. E+.

**Figure 6:**
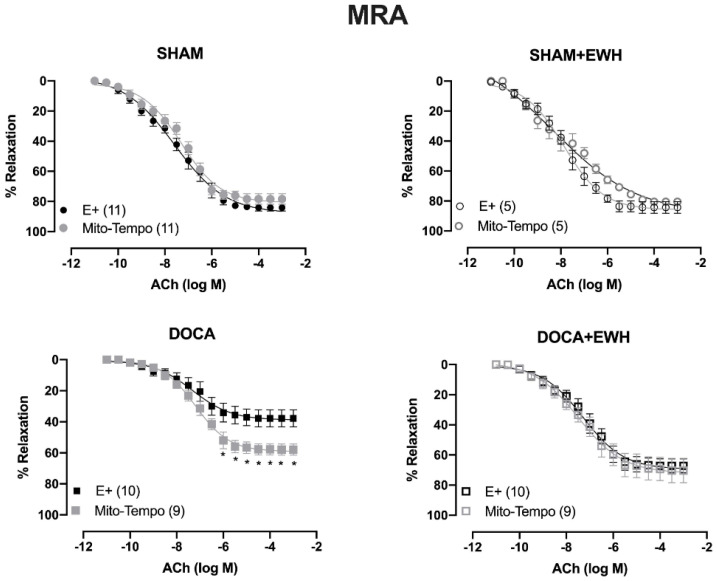

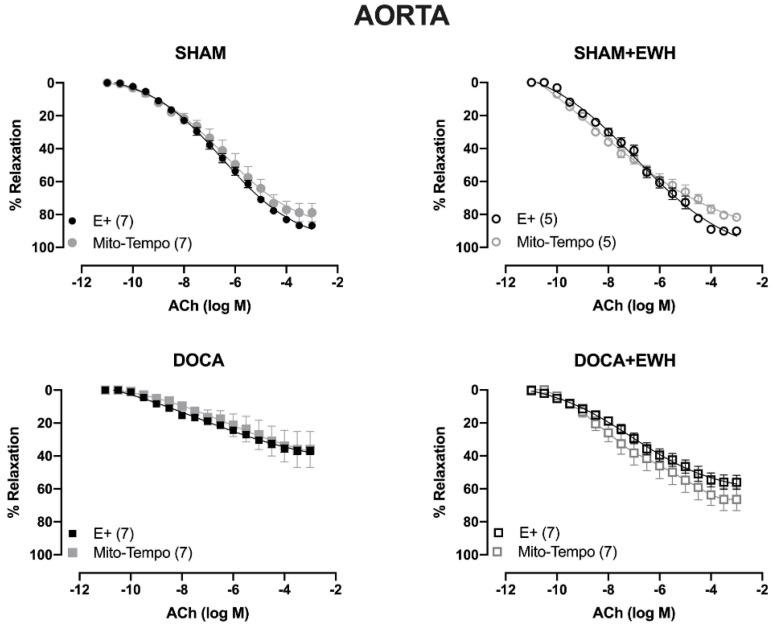
The role of EWH on mitochondrial ROS-mediated, ACh-induced relaxation in MRA and aorta of DOCA-salt rats. Concentration–response curves to ACh were obtained in MRA (Upper graphics) and aorta (Bottom graphics) segments from rats from SHAM, SHAM+EWH, DOCA, and DOCA+EWH groups before (E+) and after incubation with specific scavenger of mitochondrial superoxide, Mito-TEMPO (0.5 µmol/L). The results are expressed (mean ± SEM) as the percentage of relaxation responses in norepinephrine or phenylephrine precontracted rings. The number of animals in each group is in parentheses. Two-way ANOVA followed by Bonferroni post-test: *p* < 0.05 * vs. E+.

To investigate whether the ROS could be involved in the vascular protective action induced by EWH, the participation of the O_2_^−^ at the cellular (SOD) or mitochondrial (Mito-Tempo) level was investigated in both vessels (Figure 5 and Figure 6). In MRA, SOD incubation did not alter the relaxation induced by Ach in any group (Figure 5). However, Mito-Tempo improved the Ach-induced relaxation in the DOCA group (Figure 6). Moreover, in the MRA of SHAM, SHAM+EWH, and DOCA+EWH, Mito-Tempo did not change the ACh-induced response (Figure 6). This finding demonstrated the pivotal role of mitochondrial ROS in MRA endothelial dysfunction of the DOCA salt hypertensive model and the role of EWH in restoring this oxidative status. On the other hand, in aorta segments, only SOD (Figure 5), but not Mito-Tempo (Figure 6), was able to improve the ACh-induced relaxation in DOCA group, suggesting a dysfunctional action of the SOD in the aorta of the DOCA group and an antioxidant effect of EWH.

**Figure 7:**
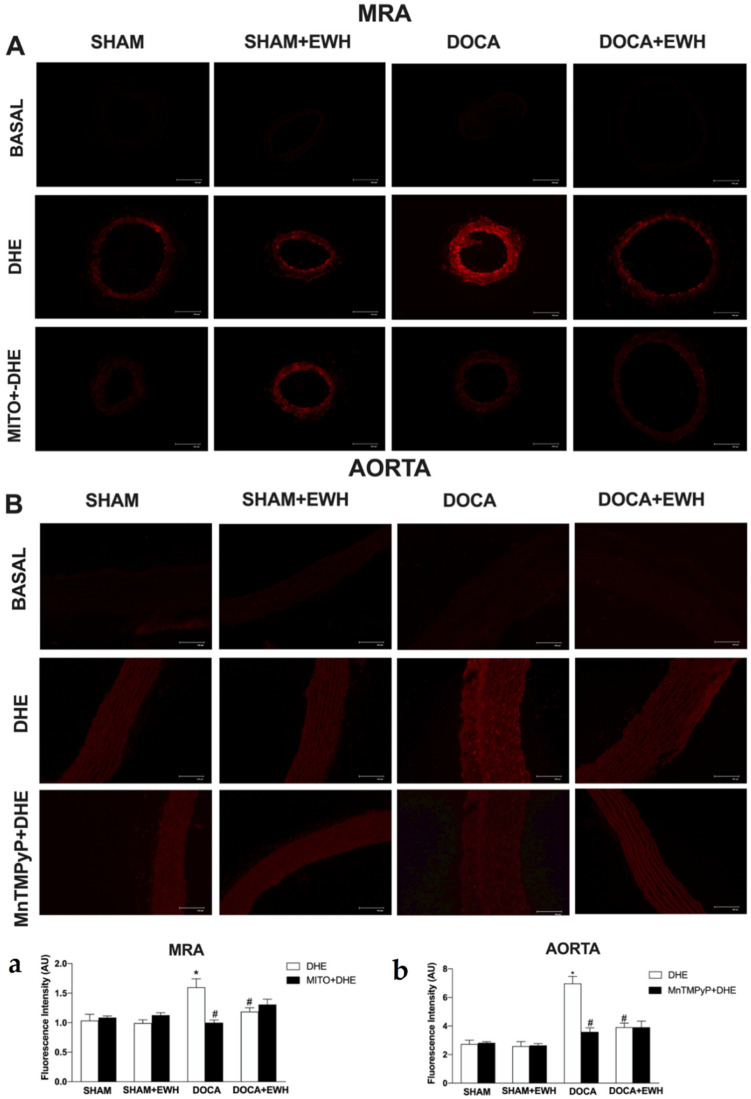
The role of EWH on O_2_^−^ production in MRA and aorta section of DOCA-salt rats. Typical images of sensitive fluorescent dye Dihydroethidium (DHE) in the absence or presence of Mito-TEMPO (0.5 µmol/L—third line panel **A**) or MnTMPyP (25 µM—third line panel **B**) in MRA and aorta sections of SHAM, SHAM+EWH, DOCA, or DOCA+EWH rats. The basal images are the negative control pictures without DHE. The histogram (**a**—MRA and **b**—Aorta) shows in white bars the DHE fluorescence and in black bars the DHE fluorescence after Mito-TEMPO or MnTMPyP incubation. The results are expressed as representative fluorescence intensity in arbitrary units. The results are expressed as mean ± SEM (n = 6). Two-way ANOVA followed by Bonferroni post-test: *p* < 0.05 * vs. SHAM; # vs. DOCA.

**Figure 8:**
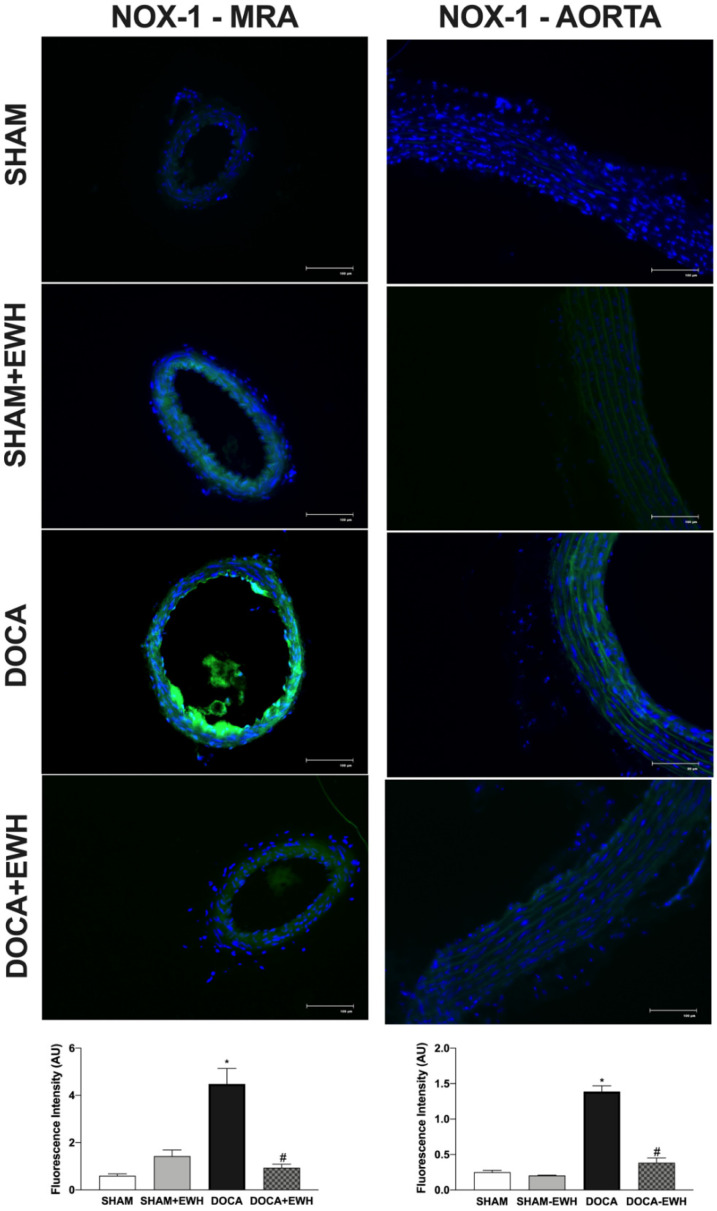
The role of EWH on NOX1 expression in MRA and aorta section of DOCA-salt rats. Representative photomicrographs (×40 magnification) and histogram of NOX1 immunofluorescence in MRA and aortic sections of SHAM, SHAM+EWH, DOCA, and DOCA+EWH arteries. Data are expressed as mean ± SEM, n = 8. The images correspond to the merge of the marking of the colors by the DAPI in blue and the NOX-1 in green. Two-way ANOVA followed by Bonferroni post-test: *p* < 0.05 * vs. SHAM; # vs. DOCA.

To corroborate these findings, in situ vascular ROS detection was evaluated. In MRA and aorta sections of the DOCA group, higher O_2_^−^ detection was observed, whereas EWH co-treatment restored this detection towards SHAM levels (Figure 7). The incubation of Mito-Tempo in MRA or MnTMPyP in aorta sections decreased the DHE fluorescence intensity in DOCA group, without additional effect in the section of the DOCA+EWH group (Figure 7). Interestingly, the NOX1 protein levels were increased in MRA and aorta of DOCA rats, and the co-treatment with EWH, once again, reduced these levels towards SHAM values (Figure 8).

**Figure 9:**
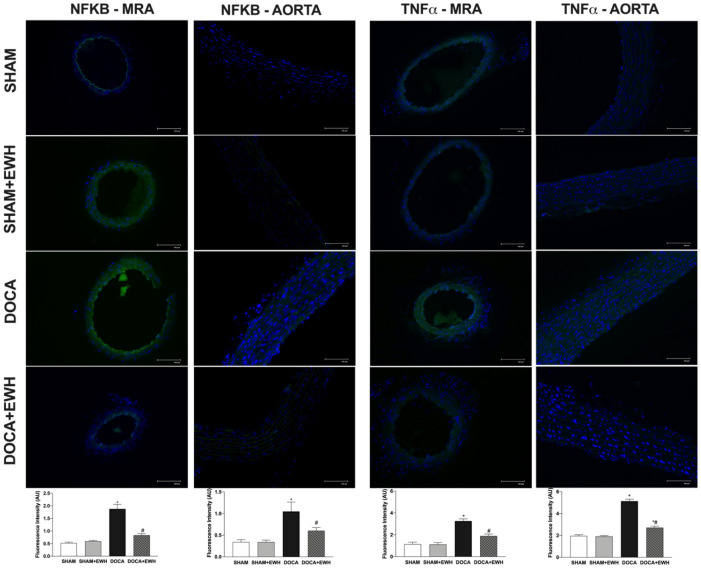
The role of EWH on NF-kB and TNFα expression in MRA and aorta section of DOCA-salt rats. Representative photomicrographs (×40 magnification) and histogram of NF-kB (left) and TNFα (right) immunofluorescence of MRA and aorta sections of SHAM, SHAM+EWH, DOCA, and DOCA+EWH arteries. Data are expressed as mean ± SEM, n = 8. The images correspond to the merge of the marking of the colors by the DAPI in blue and the NF-kB and TNFα in green. Two-way ANOVA followed by Bonferroni post-test: *p* < 0.05 * vs. SHAM; # vs. DOCA.

Vascular inflammatory mechanisms play an essential role in the development of hypertension and oxidative stress [5]; thus, we investigated the expression of NF-kB and TNFα in MRA and aorta segments. Higher NF-kB and TNFα protein levels were observed in MRA and aorta of DOCA than in the SHAM group (Figure 9), and a significant reduction was observed in arteries of DOCA co-treated with EWH (Figure 9). In addition, NF-kB inhibitor BAY 117,082 enhances ACh-induced relaxation in MRA of DOCA group compared to SHAM, but not in the SHAM, SHAM +EWH, and DOCA+EWH groups (Supplemental Figure S1), suggesting a greater activation of NF-kB in MRA segments from DOCA-salt animals that was restored by EWH co-treatment.

**Figure 10:**
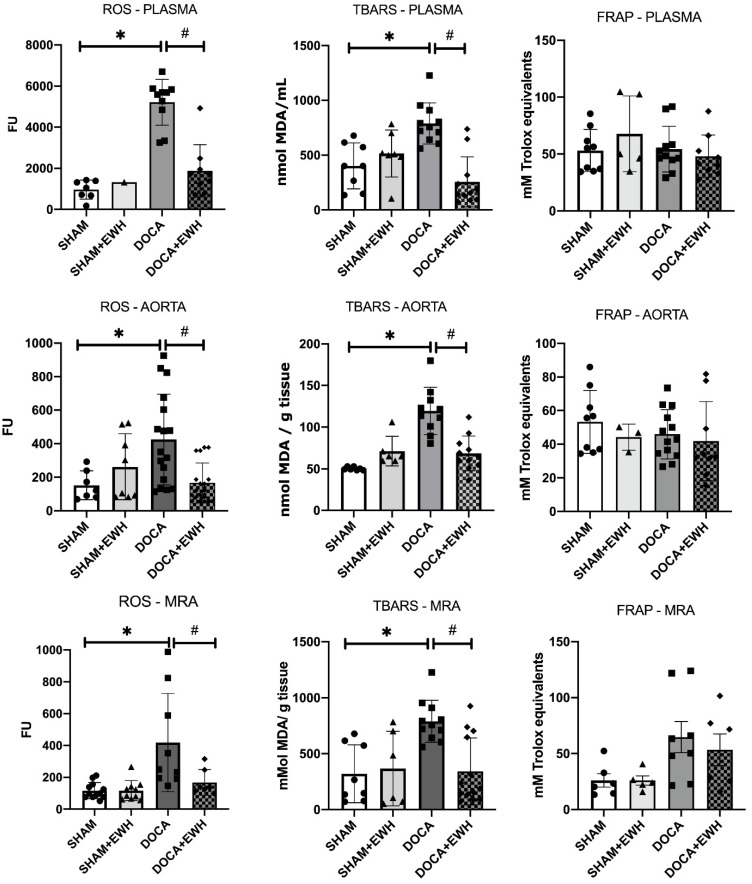
Effects of EWH on the oxidative stress (ROS and TBARS) and antioxidant (FRAP) capacity in plasma, MRA, and aorta of DOCA-salt rats. ROS, TBARS, and FRAP values were assessed in plasma, MRA, and aorta of SHAM, SHAM+EWH, DOCA, and DOCA+EWH rats. The results are expressed as mean ± SEM, and the number of animals is indicated as dots in the bars. Two-way ANOVA followed by Bonferroni post-test: *p* < 0.05 * vs. SHAM; # vs. DOCA.

Lipid peroxidation and ROS levels were increased in both plasma and arteries of the DOCA group compared to SHAM, and the co-treatment with EWH restored those parameters (Figure 10). However, DOCA-salt treatment or EWH co-treatment did not change the antioxidant capacity in plasma or arteries (Figure 10). These results suggest a redox imbalance in the DOCA group that was restored after EWH co-treatment.

**Figure 11:**
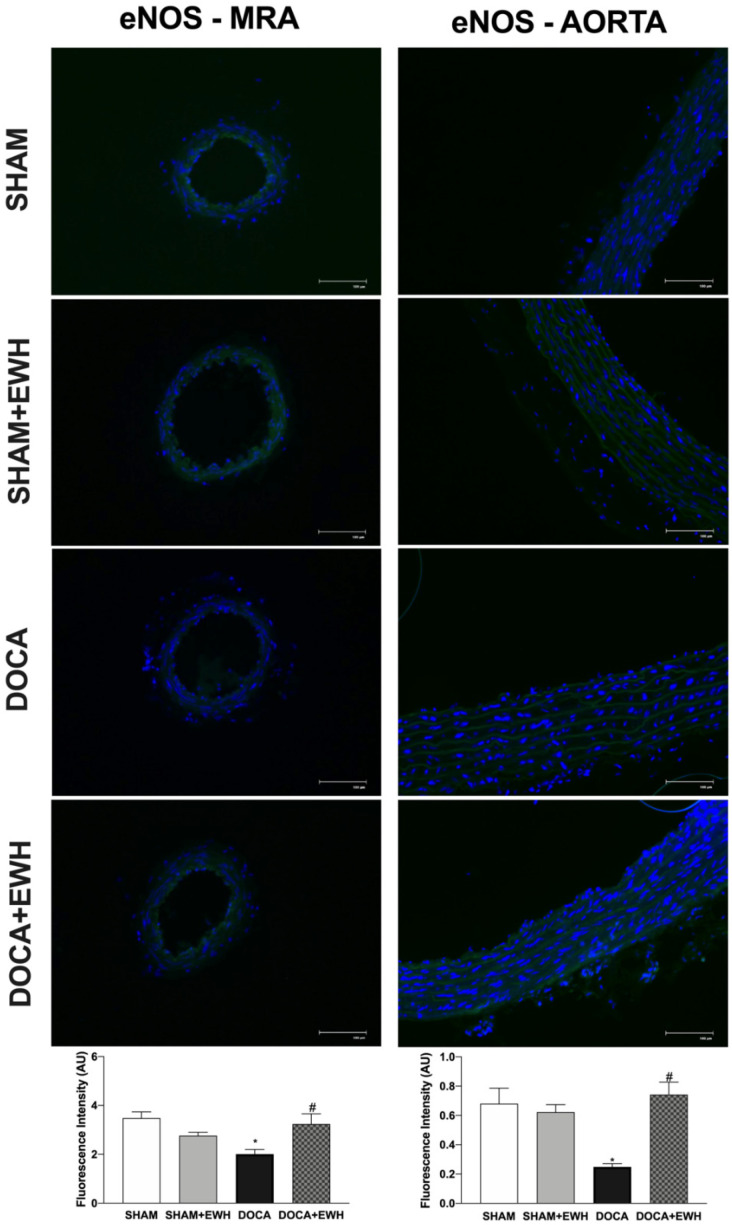
The role of EWH on endothelial NO synthase (eNOS) expression in MRA and aorta section of DOCA-salt rats. Representative photomicrographs (×40 magnification) and histogram of eNOS immunofluorescence of MRA (left panels) and aortic (right panels) sections of SHAM, SHAM+EWH, DOCA, and DOCA+EWH arteries. Data are expressed as mean ± SEM, n = 8. The images correspond to the merge of the marking of the colors by the DAPI in blue and the eNOS in green. Two-way ANOVA followed by Bonferroni post-test: *p* < 0.05 * vs. SHAM; # vs. DOCA.

Additionally, the involvement of the NO pathway was also investigated. In aorta segments, a reduced relaxation response to ACh was observed in all groups after the non-selective NOS inhibition with L-NAME (Supplemental Figure S2). However, in the DOCA group, the magnitude of the reduction was smaller compared to SHAM, and EWH co-treatment improved it (Supplemental Figure S2). To corroborate these findings, we observed a significant reduction in ACh-induced NO levels in aortic sections of the DOCA group as compared to SHAM and its recovery in the aortic section of EWH co-treated rats (Supplemental Figure S3). In addition, the eNOS expression was lower in MRA and aorta of the DOCA group than in SHAM; once again, EWH co-treatment restored eNOS expression to SHAM levels (Figure 11).

## 4. Discussion

Previously our group demonstrated that EWH has antihypertensive, vasorelaxant, antioxidant, and anti-inflammatory properties, observed in a genetic model of hypertension SHR and metals-induced hypertension [15,21,22,23,24]. In this work, for the first time, we show a significant antihypertensive effect of EWH in a model of severe hypertension induced by DOCA-salt treatment in uninephrectomized rats. The beneficial effects of EWH supplementation are associated with local (mitochondrial) and systemic antioxidant effects and anti-inflammatory action, through NF-kB and TNFα inhibition, which improves NO bioavailability and endothelium-dependent relaxation in MRA and aorta.

The relationship between hypertension and redox imbalance has been demonstrated in experimental rodent models and human hypertension [7,10,33,34]. Diets with high antioxidant content may reduce blood pressure and cardiovascular complications. In contrast, some randomized clinical and population studies have shown disappointing results, in part associated with several specific points in the trial design or dosing regimens. However, the potential pro-oxidant activity of antioxidants is a phenomenon that should not be ignored [35]. Since antioxidant food compounds have different mechanisms of action, such as activation of antioxidant enzymes, chelation of metals, blocking of lipid peroxidation, or elimination of O_2_^−^, therapeutic alternatives derived from them may be an additional tool for controlling severe hypertension [4,12]. In this sense, bioactive peptides released during food processing or after the digestion of food proteins, such as EWH, can exert different powerful biological activities [16,36]. Specifically, it is well known that peptides with antioxidant properties play a beneficial multifunctional role in the cardiovascular system and blood pressure control [4,37].

The DOCA-salt model is recognized for inducing hypertension to malignant levels and promoting vascular dysfunction, as observed in the animals in this study. Furthermore, this response was associated with an impaired synthesis or NO bioavailability and increased myogenic tone, endothelin-1 activation, ROS production by NADPH oxidase, cyclooxygenase-2, proinflammatory cytokines, NF-kB activation, and macrophage infiltration [7,10,38,39]. In addition, our study found increased ROS at systemic, cellular, and mitochondrial levels, and NF-kB and TNFα pro-inflammatory activation and reduced eNOS expression in MRA and aorta. Altogether, these results strengthen the pivotal role of vascular dysfunction in malignant hypertension and the end-organ damage observed in DOCA-salt rats. In addition, the results reinforced this model as an important model with which to study non-controlled and severe hypertension and revealed potential tools to reverse this condition.

Daily doses of EWH after the onset of DOCA-salt hypertension prevented the increase in SBP by 36% in this experimental model, as shown in Figure 2. The antihypertensive effect of EWH was previously demonstrated. EWH supplementation reduced and prevented the development of hypertension in SHR rats [14,15]. In addition, in animals exposed to toxic metals such as mercury and aluminum, EWH can reduce blood pressure to control levels. However, in this metal’s exposure models, the blood pressure levels were within normal limits [21,23] or in stage 1 of hypertension [2,22]. Moreover, in rats exposed to high concentrations of cadmium, which showed hypertension at high levels, EWH also reversed high blood pressure levels towards those of the control animals [24]. An essential point on which those results differ from those observed in the present study is that in animals exposed to toxic metals, the treatment was performed as a preventive tool, during the development of hypertension. By contrast, in the present study, the treatment was performed as a therapeutic tool, when hypertension was established.

As we described above, hypertension in the DOCA-salt model impairs vasodilator responses [7,10,40,41,42], and these vascular effects are strongly related to systemic and locally promoted oxidative stress and inflammation [7,10,43]. However, until now, there have been no studies in this model evaluating the effect of dietary EWH supplementation on vascular damage. The direct vasorelaxant effect of EWH and its isolated bioactive peptides were previously described in conductance and resistance arteries [15,16,19,21,22,23,24]. Fujita et al. (1995) isolated ovokinin peptide, whose amino acid sequence (Phe-Arg-Ala-Asp-His-Pro-Phe-Leu) was also identified in this EWH. Ovokinin showed a significant vasorelaxant response in the MRA of SHR rats [44]. Furthermore, other isolated peptide sequences (RADHPFL, RADHP, YRGGLEPINF, RDILNQ) of EWH also showed vasodilator properties in MRA, associated with increased NO synthesis, probably due to its amino acid composition and mainly due to the N-terminal position of Arg or Tyr [19]. In the present study, the improvement in ACh-induced endothelium-dependent relaxation in MRA and aorta of the DOCA-salt rats supplemented with EWH was due to the increase in endothelial function, as NO donor did not change its response among groups. The present results agree with previous results using models of exposure to different metals, in which the protective effect of EWH on the endothelium was observed in resistance and conductance vessels [21,22,23,24].

The bioavailability of NO is an important modulating factor of vascular vasodilator responses [7,45]. The present results demonstrated that the lower eNOS expression observed in MRA and aorta was restored towards the SHAM levels. In addition, using aorta segments as a model that represents the pivotal role of NO for endothelium-dependent relaxation, the present results also demonstrated that EWH supplementation in DOCA-salt rats recovered the NO-dependent participation in the ACh-induced relaxation, suggesting that EWH supplementation could improve NO bioavailability. Likewise, EWH restored the decreased NO levels in the arteries of rats exposed to mercury [23], and this effect was related to its capacity to increase NO release by enhancing eNOS activity [37]. Furthermore, the relaxation promoted by the RADHPFL, a peptide included in EWH, was endothelium-dependent and mediated by NO production via bradykinin B1 receptor activation [17]. In SHR rats treated with ovotransferrin-derived peptide, one of the main proteins present in raw egg white, NO levels were preserved in MRA [46].

NO bioavailability is the balance between NO synthesis and degradation. Thus, the restored endothelial function induced by EWH supplementation in MRA and aorta of DOCA-salt rats may be related to the antioxidant profile of EWH. This mechanism was confirmed in the present study by the reduction of oxidative stress (MDA and ROS) parameters in plasma and arteries, and by functional and immunohistochemical data. The antioxidant capacity of EWH and its bioactive peptides has been also previously described in models of genetic obesity [47] and metabolic syndrome [48]. In those studies, the antioxidant properties of EWH are related to some peptides, particularly by the Tyr-Ala-Glu-Glu-Arg-Tyr-Pro-Ile-Leu (YAEERYPIL) sequence, which has high radical scavenging activity.

An important source of O_2_^−^ at the vascular level is the activation of the NADPH oxidase enzyme, related to the increase of NOX1 expression and activation [7,49,50,51]. In the present study, we observed that EWH was able to restore NOX-1 expression and O_2_^−^ production in MRA and aorta of the DOCA-salt group. In line with these results, previously we reported that EWH also prevents the increase in ROS arising from the activation of NADPH oxidase and activation of NOX-1 and/or NOX-4 in animal exposure to heavy metals [21,22]. In the last two decades, the vital role of mitochondria in maintaining vascular homeostasis and ROS production has been highlighted [52,53]. In addition, mitochondrial O_2_^−^ generation promoted vascular dysfunction in resistance and conductance arteries of the DOCA-salt model was described [8]. In part, that endothelial dysfunction is related to depletion of Sirt3 (a protein essential for mitochondrial health) and mitochondrial ROS generation [45]. In our study, MRA O_2_^−^ generation was derived from two distinct sources, NADPH oxidase and mitochondria, and Mito-Tempo incubation and EWH supplementation restored the in situ and ex vivo mitochondrial ROS production observed in DOCA-Salt rats. Previously, EWH has described increasing mitochondrial DNA gene expression in brown fat tissue in a metabolic syndrome model [54]. However, the present study is the first that demonstrated EWH scavenging action on mitochondrial O_2_^−^ generation in MRA; by this mechanism, EWH contributes to the restoration of endothelial function.

Another important mechanism related to excessive ROS production in the DOCA-salt model is the activation of transcription factors, which seems to be related to an inflammatory response and endothelial dysfunction [4,15,49]. Moreover, TNFα deficiency improved endothelial function and cardiovascular injury in hypertension [55]. Our study also demonstrated that in MRA and aorta of DOCA-salt hypertensive rats, increased NF-kB and TNFα expression by immunofluorescence and EWH supplementation restored the pro-inflammatory markers towards SHAM levels. In addition, we also observed the restoration of endothelial dysfunction in MRA of DOCA-salt rats by the blockage of NF-kB activation and no additional effect in MRA of DOCA-salt rats supplemented with EWH. In vessels exposed to metals, the anti-inflammatory role of EWH was previously described and related to cyclooxygenase-2 inhibition [21,22,23,24]. In addition, EWH was effective in reducing the systemic plasma levels of TNFα in obese rats [20]. Thus, the present results also show that by its anti-inflammatory effect and inhibition of NF-kB and TNFα expression, EWH may improve endothelial dysfunction in arteries of DOCA-salt rats.

## 5. Conclusions

The EWH supplementation in DOCA-salt rats, when the hypertension was stabilized, restored the mitochondrial and cellular ROS production, NF-kB activation, and TNFα pro-inflammatory levels, improving the eNOS expression and endothelial function in MRA and aorta and reducing blood pressure levels. EWH could be used as a natural functional food ingredient or as an adjuvant supplement in the treatment of vascular dysfunction related to malignant hypertension.

## Data Availability

Data is contained within the manuscript or supplementary material.

## Supplementary Materials

The following supporting information can be downloaded at: https://www.mdpi.com/article/10.3390/antiox11091713/s1, Figure S1: The role of EWH in NF-κB-induced impairment on acetylcholine (ACh) relaxation in MRA of DOCA-salt rats; Figure S2: The role of EWH in NO-mediated acetylcholine (ACh)-induced relaxation in the aorta of DOCA-salt rats; Figure S3: NO production is recovered by EWH treatment in aortic sections of DOCA-salt rats.
