# Supplementary material for: ROS Suppression by Egg White Hydrolysate in DOCA-Salt Rats—An Alternative Tool against Vascular Dysfunction in Severe Hypertension
Source: Antioxidants (Basel). 2022 Aug 30;11(9):1713. doi: 10.3390/antiox11091713 (PMC9495903; doi:10.3390/antiox11091713)
Supplement: Supplementary file 1 [file antioxidants-11-01713-s001.zip › antioxidants-1861472-supplementary.pdf]

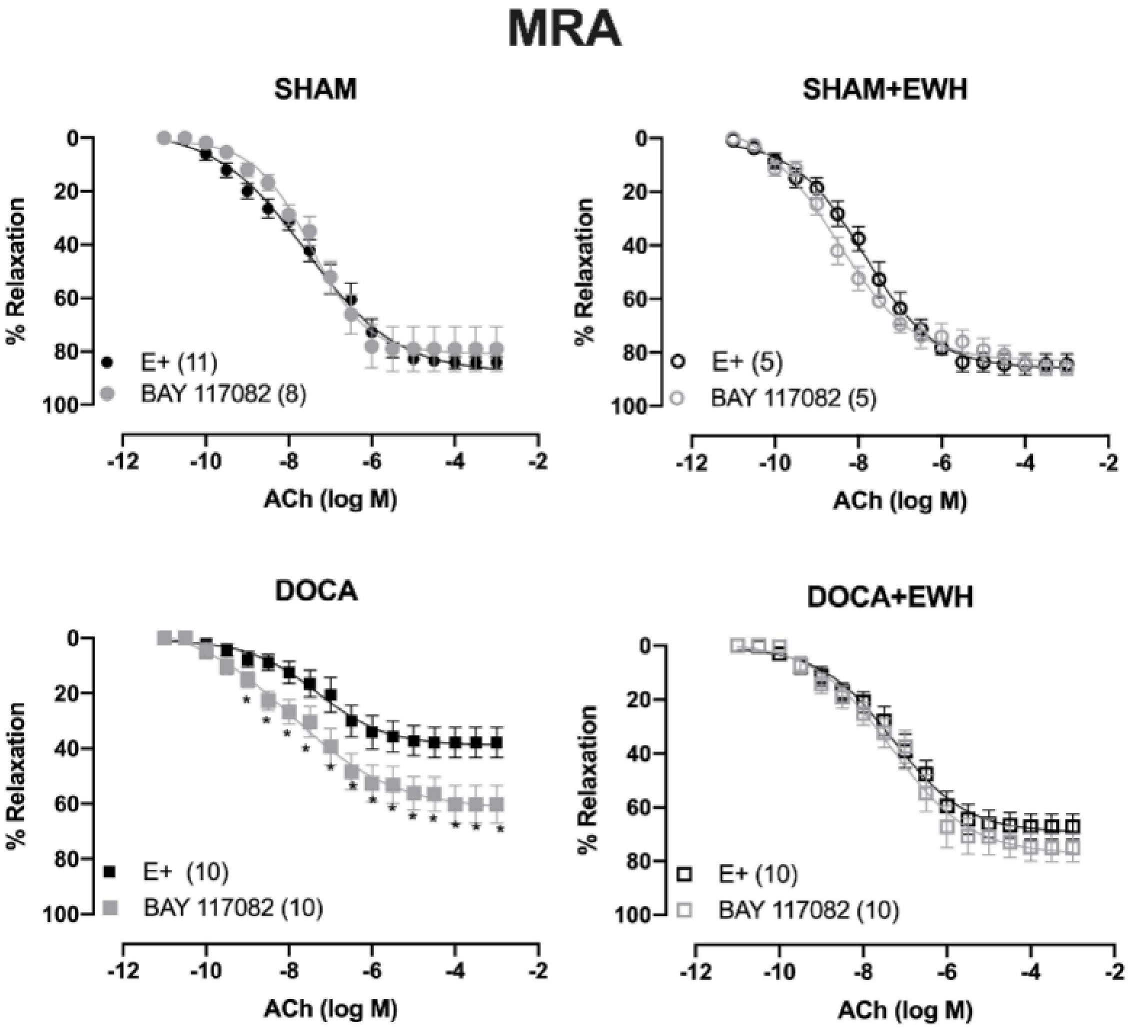

Figure S1. The role of EWH in NF-κB-induced impairment on acetylcholine (ACh) relaxation in MRA of DOCA-salt rats. Concentration-response curves to ACh were obtained in MRA from rats from SHAM, SHAM+EWH, DOCA, and DOCA+EWH groups before (E+) and after incubation with NF-κB inhibitor (BAY 117082. 5 μM). The results are expressed (mean ± SEM) as the percentage of relaxation responses to norepinephrine precontracted rings. The number of animals in each group is in parentheses. Two-way ANOVA followed by Bonferroni post-test: $p < 0.05$ **vs.* E+.

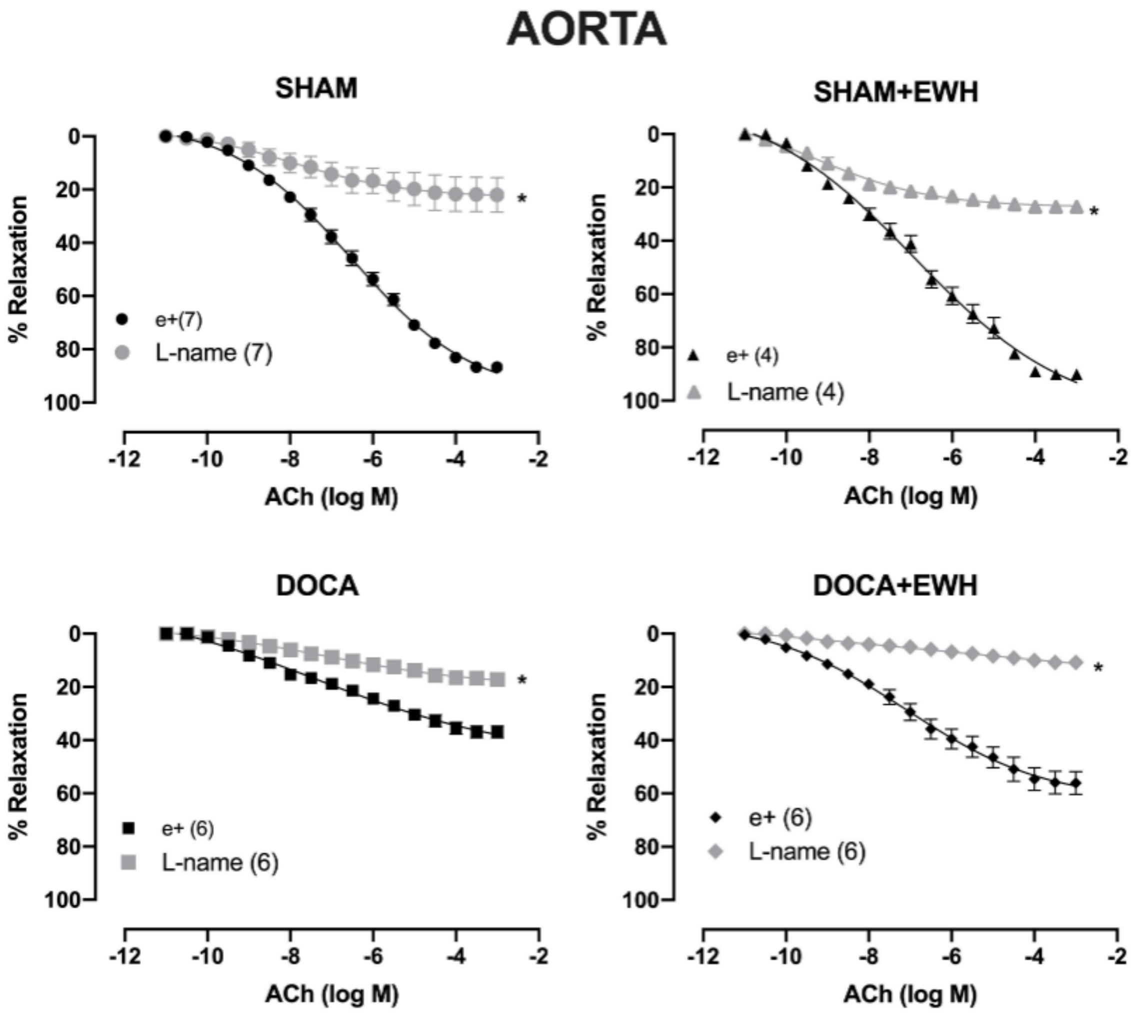

Figure S2. The role of EWH in NO-mediated acetylcholine (ACh)-induced relaxation in the aorta of DOCA-salt rats. Concentration-response curves to ACh were obtained in the aorta of rats from SHAM, SHAM+EWH, DOCA, and DOCA+EWH groups before (E+) and after incubation with a non-selective inhibitor of NOS (L-NAME, 100 μM). The results are expressed (mean ± SEM) as the percentage of relaxation responses to phenylephrine precontracted rings. The number of animals in each group is in parentheses. Two-way ANOVA followed by Bonferroni post-test: $p < 0.05$ **vs.* E+.

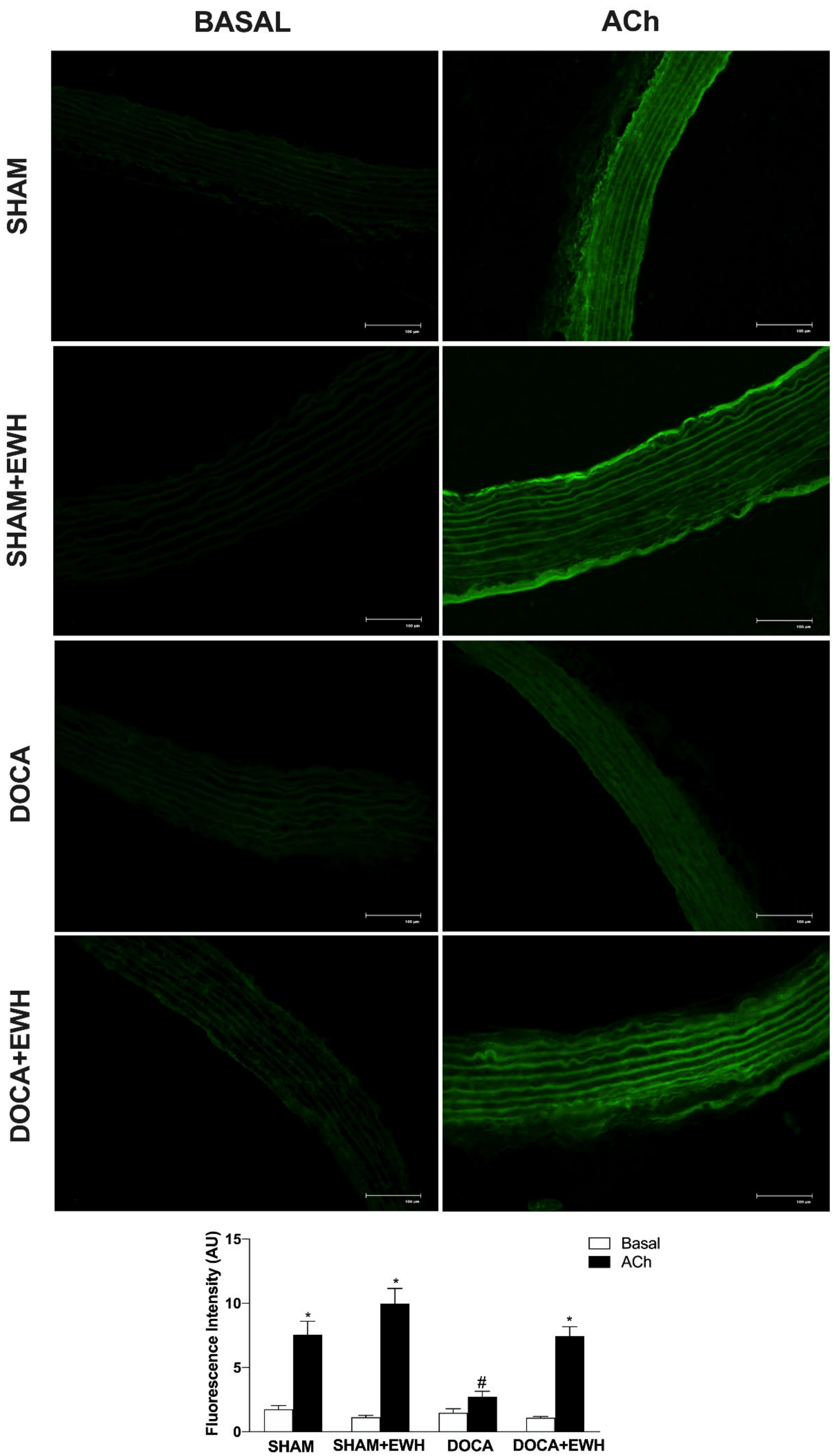

BASAL
ACh
SHAM
SHAM+EWH
DOCA
DOCA+EWH
Fluorescence Intensity (AU)
15
10
5
0
Basal
ACh
*
*
#
*
SHAM
SHAM+EWH
DOCA
DOCA+EWH

Figure S3: NO production is recovered by EWH treatment in aortic sections of DOCA-salt rats. Representative fluorographs of transverse sections of aorta loaded with DAF-2 in the absence (Basal, left panel) and presence of ACh (100 μmol/L, right panel) in SHAM, SHAM+EWH, DOCA, and DOCA+EWH groups. Bar graphs show the quantified NO production measured as DAF-2 fluorescence in sections of the aorta in basal condition (white bar) and after ACh stimulation (black bar). The values are presented as the mean ± SEM. N=4. Significance was assessed with a two-way ANOVA: $p < 0.05$ *_vs._ basal condition, # _vs._ SHAM.
